# Postoperative Extra-Cranial Metastasis of Glioblastoma: A Case Report

**DOI:** 10.30699/ijp.2020.122780.2349

**Published:** 2020-10-10

**Authors:** Arezoo Eftekhar Javadi, Hedieh Moradi Tabriz, Atieh Zandnejadi

**Affiliations:** 1 *Department of Anatomical and Surgical Pathology and Laboratory Medicine, Sina Hospital, Tehran University of Medical Sciences, Tehran, Iran*

**Keywords:** Brain Tumor, Glioblastoma, Metastasis, Pelvic Cavity

## Abstract

Glioblastoma (WHO grade IV) is the most common malignant tumor of neural tissues in adults as a primary tumor. Because of blood brain barrier and short median survival of patients with glioblastoma, metastasis of this tumor is very rare. A 46-year-old man was admitted to Sina hospital with chief complaint of headache and visual impairment. After neuro-radiologic evaluation the patient underwent surgery. Pathologic examination of the tumor confirmed the diagnosis of glioblastoma multiforme. Cytogenetic study of the tumor cells confirmed GBM IDH1 wild type with TERT mutation and EGFR amplification. Two months after surgical resection, the tumor recurred with involvement of the dura matter. After the second operation, metastasis to the pelvic cavity and cervical lymph node was found. Almost all cases of glioblastoma metastasis had undergone surgery or any manipulation; this fact suggests that iatrogenic intra-vascular seeding of tumor cells at the time of resection and disruption of blood brain barrier could cause extra-neural metastasis.

## Introduction

Glioblastoma (WHO grade IV) is the most common neurologic malignant tumor of neural tissue in adults as a primary tumor (1). It shows aggressive behavior with median survival of about one year despite surgical resection, chemotherapy and radiation (2). Blood brain barrier mechanism keeps other organs safe from metastasis of CNS primary tumors. Because of BBB and short median survival of patients with glioblastoma, its metastasis is very rare (3). While direct extension and recurrence are typical, the incidence of extra-neural metastasis of glioblastoma is 0.2% (4) and mainly occur in men (5). First case of disseminated glioblastoma was reported by Davis in 1928 (2).

Almost all cases of glioblastoma metastasis had undergone surgery or any manipulation; this suggests that iatrogenic intra-vascular seeding of tumor cells at the time of resection could disrupt blood brain barrier and cause extra-neural metastasis (6). Although researches showed that 10% of metastatic GBM had no correlation with any surgical intervention (1).

Here we presented a 46-year-old man who underwent resection for glioblastoma. After two months a second operation was performed due to tumoral recurrence and 6 months after that metastasis to the pelvic cavity and cervical lymph node was found.

##  Case Report

A 46-year-old man was admitted to our hospital with chief complaint of headache and visual impairment in July 2018. Brain MRI of showed centrally non-enhancing subcortical brain lesion with ring enhancement in favor of glioblastoma. Therefore, the patient underwent surgical resection.

**Fig.1:**
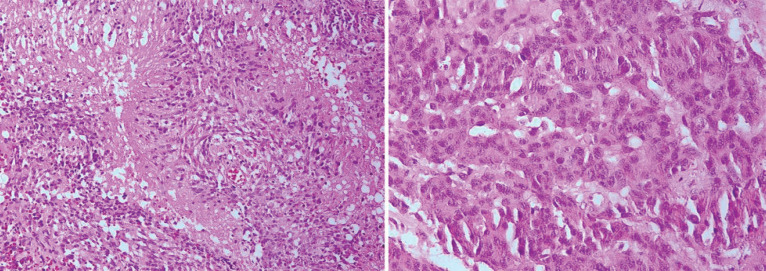
Nuclear pseudo-palisades and pleomorphism of tumor cells

Microscopic evaluation showed a neoplastic tissue composed of dysplastic cells with enlarged hyperchromatic and pleomorphic nuclei. Frequent mitotic activities were seen. The stroma showed a vascular proliferation, thrombosis and extensive area of necrosis. Nuclear pseudo-palisades, were also noted. The diagnosis with morphologic features reported as glioblastoma multiforme (WHO GRADE IV) (Figure 1). Cytogenetic study of the tumoral cells confirmed GBM, IDH1 wild type with TERT mutation and EGFR amplification.

**Fig. 2:**
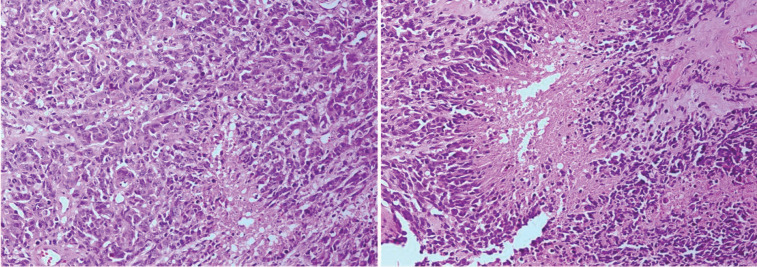
Microscopic feature of the recurrent tumor showed the same morphology as first tumor, including nuclear pleomorphic, nuclear pseudo-palisades and necrosis

Two months after surgical resection due to local recurrence, the patient underwent a second operation and resection of the tumor with involved dura mater. Microscopic evaluation of second specimen was the same as the first with addition of dura mater involvement (Figure 2).

**Fig. 3:**
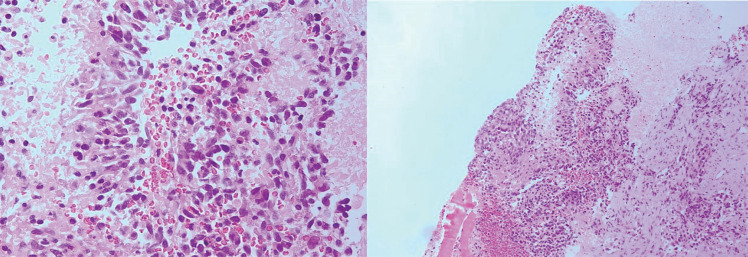
Microscopic features of the pelvic mass core needle biopsy

**Fig. 4:**
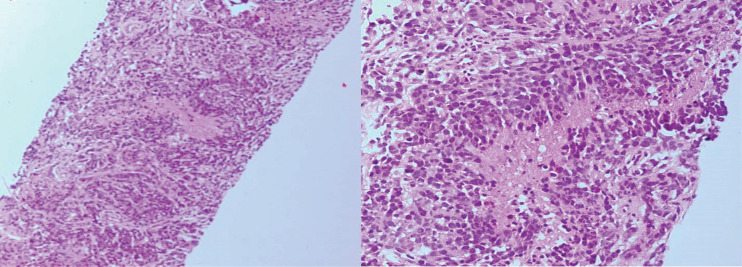
Microscopic features of the cervical lymph node core needle biopsy

However, six months after the second surgery the patient underwent core needle biopsy of pelvic mass and suspicious cervical lymph node found in examinations. Both specimens of core needle biopsy revealed histological features the same as the resected brain tumor including neoplastic growth of malignant astrocytic cells with nuclear atypia and pleomorphism as well as giant cells in background of fibrotic and palisading tissue. Mitosis were also present (Figures 3 and 4)

**Fig. 5:**
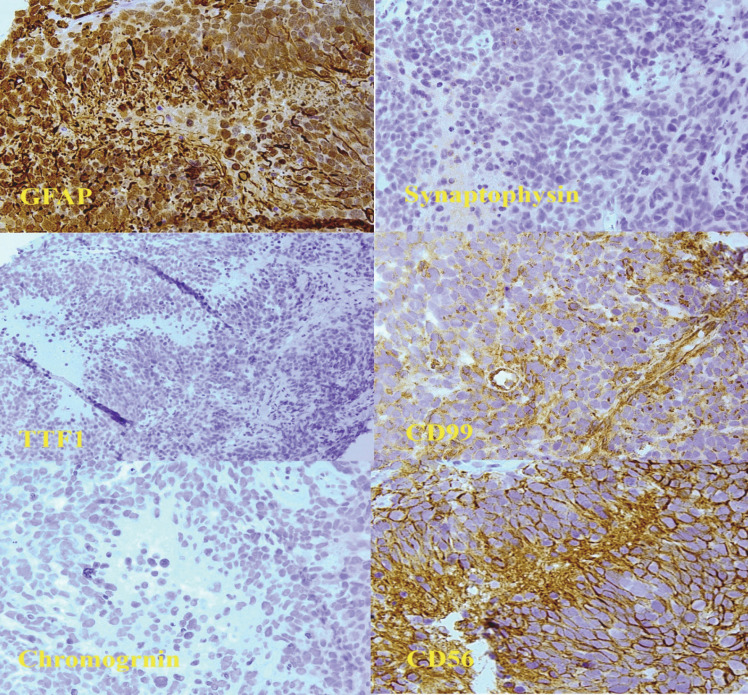
IHC panel for the pelvic mass evaluation which showed positive staining for GFAP and negative for Synaptophysin. Negative staining for TTF-1 and non-specific staining for CD99 were also noted. Two later pictures show positive staining for CD56 and negative staining for chromogranin

Immunohistochemical studies of the pelvic mass confirmed the diagnosis, showed positive results for GFAP, CD56 and WT1 stains and negative results for Synaptophysin, Chromogranin and TTF-1 stains (Figure 5). Unfortunately, the patient died less than one year after diagnosis.

## Discussion

Glioblastoma is one of the most difficult tumors to treat in neurosurgical management. The tumor mortality is high and most patients die due to oncothlipsis, intracranial hypertension or other complications. The median survival is about one year (7).

Blood brain barrier, lack of true lymphatic system and low survival rate are considered as some reasons to explain why distant metastasis of this tumor is very rare (8). However, among all glioma tumors, glioblastoma is the most common tumor with extra- cranial metastasis (41.4 %) (9). First case of glioblastoma metastasis to extra neural tissue was reported by Davis in 1928 (6).

Younger and healthier patients are more prone to present with extracranial metastases, due to longer overall survival (OS) compared to elderly GBM patients with multiple chronic illnesses. (10)

There are other case reports suggesting lymph nodes, lung, spinal cords and bone as the most common metastatic sites (11,12). Liver metastasis seems to have more poor outcomes than other sites (13). Here we reported a rare location of metastasis to the pelvic cavity.

The median time of detection of metastasis has been reported as about 8.5 months after diagnosis of GBM and mortality occurs about 1.5 month after metastasis as there is no effective treatment regimen. 

Metastatic GBMs are in two different patterns including neuroaxial and systemic. Although in neuroaxial metastasis debulking might be helpful, in the systemic distal metastasis as in our case, organ specific consideration should be made for adjuvant therapy (14).

In this case, distant metastasis occurred about 8 months and the time of death was within 2 months. Our patients had many risk factors of metastasis including two surgeries, radiation and not getting chemotherapy and the most important aggressive behavior of primary GBM- IDH1 wild type/TERT+/EGFR amp+ in cytogenetic evaluation (15).

## Conclusion

Glioblastoma is one of the hardest tumor to treat in neurosurgical management. Blood brain barrier, lack of true lymphatic system and low survival mode this tumor very rare to metastasis. Almost all cases of glioblastoma metastasis had undergone surgery or any manipulation; this fact suggests that iatrogenic intra-vascular seeding of the tumoral cells at the time of resection and disruption of blood brain barrier could cause extra-neural metastasis.
